# Diffuse Hepatic Epithelioid Hemangioendothelioma Developed in a Patient with Hepatitis C Cirrhosis

**DOI:** 10.1155/2014/694903

**Published:** 2014-09-08

**Authors:** Pedro W. Baron, Thomas Amankonah, Robert F. Cubas, Arputharaj H. Kore, Arvand Elihu, Michael E. de Vera, Mia C. N. Perez

**Affiliations:** ^1^Transplantation Institute, Loma Linda University Medical Center, 25865 Barton Road, Suite 101, Loma Linda, CA 92354, USA; ^2^Department of Surgery, Loma Linda University Medical Center, 25865 Barton Road, Suite 101, Loma Linda, CA 92354, USA; ^3^Departments of Pathology and Laboratory Medicine, Loma Linda University Medical Center, 25865 Barton Road, Suite 101, Loma Linda, CA 92354, USA

## Abstract

Hepatic epithelioid hemangioendothelioma (HEHE) is an infrequent vascular tumor of endothelial origin that primarily occurs in women in the mid-fifth decade of life without underlying chronic liver disease or cirrhosis. Liver transplant should be the first-line of therapy in patients with large or diffuse unresectable tumors even in the presence of metastatic disease due to the favorable long-term outcome. We report the case of a 48-year-old female who complained of abdominal pain and weight loss. She has a history of cirrhosis secondary to chronic hepatitis C (HCV) and was treated with interferon and ribavirin with sustained virological response. Her work-up revealed multiple confluent infiltrating bilobar liver masses diagnosed as HEHE. She underwent a successful liver transplant without evidence of recurrent HCV infection. She developed cervical spine (C4-C6) HEHE metastases 4 years after transplant. She underwent surgical resection and local radiotherapy after resection with good clinical response. To the best of our knowledge, this is the first report of HEHE that developed in a patient with HCV cirrhosis successfully treated with antiviral therapy before transplant and liver transplant with good allograft function without evidence of recurrent liver tumor or HCV infection but developed metastases to the cervical spine 4 years after transplant.

## 1. Introduction

Hepatic epithelioid hemangioendothelioma (HEHE) is an infrequent vascular tumor of endothelial origin, not associated with cirrhosis, with a histological appearance intermediate between hemangioma and angiosarcoma [1].

HEHE has an incidence of less than 0.1 per 100,000 population [2], accounts for less than 1% of all hepatic malignancies, and shows a 3 : 2 female preponderance [3]. HEHE was first described in 1982 by Weiss and Enzinger [4]. Ishak et al. reported the first series of 32 primary hepatic hemangioendotheliomas in 1984 [5]. There are no clearly established risk factors for its development; however, association with oral contraceptive pill intake, vinyl chloride exposure, major hepatic trauma, and viral hepatitis has been implicated [1, 3]. Generally, HEHE behaves as a low-grade malignant tumor with a slowly progressive phenotype [6]. Its clinical presentation is nonspecific and can vary from no symptoms to overt hepatic failure and death [7].

Patients with HEHE are often candidates for liver transplant (LT) as the disease is usually multifocal at diagnosis and it is also resistant to both chemotherapy and radiotherapy.

## 2. Case Report

**Figure 1:**
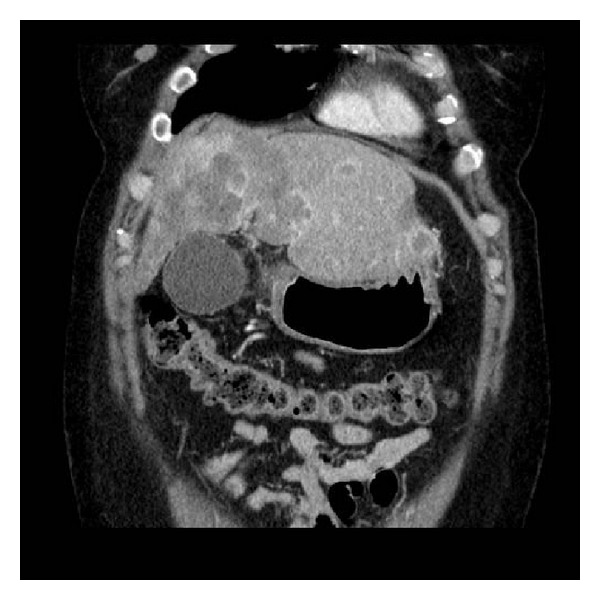
CT scan of the abdomen and pelvis.

We report the case of a 48-year-old Caucasian female who complained of abdominal pain and 30-pound-weight loss over a three-month period. She had a history of chronic hepatitis C (HCV), genotype 3a, that was treated with peginterferon alpha and ribavirin with HCV RNA negative after 8 weeks of treatment. She continued HCV therapy for about 24 weeks until transplant. On initial physical examination, patient was anicteric but with increased liver span of 14 cm by dull percussion which is consistent with hepatomegaly. Additional pretransplant work-up showed normal hemoglobin and serum alpha-fetoprotein (AFP), low platelet count, and mildly elevated serum aminotransferase levels. Abdominal and pelvic computerized tomography (CT) scan showed multiple confluent infiltrating bilobar low density liver masses and cirrhosis (Figure 1). A percutaneous liver core biopsy was performed. The initial histopathologic interpretation was inconclusive. Following an additional positive factor VIII-related antigen stain, the diagnosis of HEHE was rendered. She was listed for LT following a Liver Regional Review Board approval with a model for end-stage liver disease score exception of 22 and gained additional points corresponding to 10% waiting-list mortality every 3 months. Patient underwent a successful LT 13 months later.

**Figure 2:**
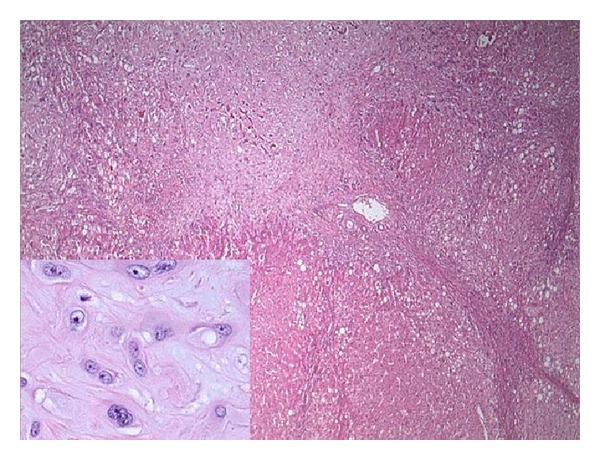
Photomicrograph of explanted liver. Transition zone between HEHE (upper-left corner) and cirrhosis. Inset is high-power view of HEHE showing epithelioid cells with intracytoplasmic lumina.

The explanted liver showed hepatomegaly (2,480 grams in weight) and a micronodular capsular surface. Cut sections revealed cirrhosis and multiple, firm, tan-white nodules measuring up to 2.2 cm in greatest diameter. Histopathologic examination confirmed multifocal HEHE in the background of cirrhosis and steatosis (Figure 2). The typical HEHE features, consisting of anastomosing cords of epithelioid cells, mostly with intracytoplasmic lumina, in a hyalinized and myxoid stroma, were seen (Figure 2 inset). There were also atypical features with a solid growth pattern and spindle cell cytology. A positive CD31 stain again confirmed the vascular endothelial nature. The tumor additionally showed extracapsular spread with direct extension to perihilar lymph nodes and hilar vessels. Proliferation marker Ki-67 showed differential staining with less than 5% positivity in the typical areas and 10–15% positivity in the atypical areas.

Her immunosuppressive therapy consisted of methylprednisolone IV bolus after hepatectomy was completed and then tacrolimus and prednisone as maintenance therapy. The patient was discharged home with excellent allograft function on postoperative day 8. She has not had an episode of acute rejection. Prednisone was discontinued 6 months after transplant. She underwent total right shoulder replacement due to severe degenerative arthritis and chronic pain without complications 15 months after transplant. She developed cervical spine (C4-C6) HEHE metastases 4 years after transplant. She underwent surgical resection and local radiotherapy after resection. Her allograft function is normal and she has no evidence of recurrent HCV infection. This report was completed after obtaining written authorization from the patient, as per our standard Institutional Review Board policy.

## 3. Discussion

To the best of our knowledge, this is the first report in English literature of a case of HEHE that developed in a patient with HCV cirrhosis, RNA-negative following pretransplant therapy and treated with LT, without HCV recurrence for more than 48 months after transplant. However, she developed metastatic cervical spine disease 4 years after transplant treated with resection and radiotherapy with good clinical response.

Several factors, including viral hepatitis, have been implicated in the pathogenesis of HEHE. Makhlouf et al. reported three HEHE patients who tested positive for hepatitis B virus and another three HEHE patients who tested positive for hepatitis C virus. None of the six patients had cirrhosis at the time of the HEHE diagnosis [8]. Grotz et al. reported 30 patients with HEHE, none of whom had underlying chronic liver disease or cirrhosis [1]. Mehrabi et al. stated that HEHE, in contrast to many other types of primary liver tumor, does not arise in a background of chronic liver disease [9–12]. No clear association was found between HEHE and HCV cirrhosis in patients who underwent LT between 1990 and 2010 after reviewing the Organ Procurement and Transplantation Network (OPTN) database^∗^. Two LTs were done for a vascular tumor of the liver as a diagnosis group that includes HEHE, hemangiosarcoma, and angiosarcoma with a secondary diagnosis of HCV cirrhosis. It was not possible to determine whether these two patients had HEHE and HCV cirrhosis.

Most patients with HEHE seek medical attention after the tumor burden has become large enough to cause symptoms [13]. Upper abdominal pain or discomfort appears to be the most frequent symptom. Although CT scan may detect the lesions, no imaging features of HEHE are pathognomonic [1].

Histopathologic examination is the only way to confirm the diagnosis of HEHE. Distinguishing HEHE from other liver tumors is vital, because the 5-year survival rate of HEHE has been reported to be greater than 55%, which is favorable compared to other primary liver malignant neoplasms [14].

There is no standard treatment for HEHE due to its infrequent incidence. Either surgical resection or LT has been generally recommended as curative treatment for this disease [1, 15]. HEHE is usually multifocal and therefore not suitable for partial hepatectomy in about 87% of the cases [12]. It also seems to be resistant to both chemotherapy and radiotherapy.

Mehrabi et al. reviewed 434 patients treated for HEHE since 1984 and concluded that LT is the treatment of choice because of the hepatic multicentricity with 96% and 54% patient survival rates at 1 and 5 years, respectively [12]. Lerut et al. reported that the outcome of LT as a treatment for HEHE was excellent with 83% and 72% patient survival rates at 1 and 5 years, respectively [16]. Rodriguez et al. analyzed 110 patients from the UNOS database who underwent LT between 1987 and 2005. These data indicated that survival after LT was favorable for all patients, including patients with diffuse intrahepatic tumor, with 1-year and 5-year patient survival rates of 80% and 64%, respectively [13]. Preexisting extrahepatic disease spread and lymph node involvement are not contraindications to LT. However, microscopic and/or macroscopic vascular invasion significantly influence post-LT survival [16–18].

Although these patients achieve excellent post-LT outcomes, there is very little data regarding tumor markers that can further direct chemotherapy in HEHE to prevent disease recurrence [18]. Tumor markers (i.e., AFP, carcinoembryonic antigen/CEA, and cancer antigen/CA 19-9) are usually in the normal range; however, vascular endothelial growth factor expression was identified in all specimens from a cohort of six patients with HEHE at a single center (100%) [13, 18].

If radiologic characteristic of hepatocellular carcinoma (HCC) is not present, high index of suspicion is necessary to rule in this very rare liver tumor in the background of hepatitis C cirrhosis, granting that HCC is the most common malignancy by far.

In conclusion, to the best of our knowledge, this is the first report of HEHE that developed in a patient with HCV cirrhosis successfully treated with antiviral therapy before transplant and liver transplant with good liver allograft function without evidence of recurrent liver tumor or HCV infection but developed metastases to the cervical spine 4 years after transplant.

## Abbreviations

HEHE: Hepatic epithelioid hemangioendothelioma
LT: Liver transplant
HCV: Hepatitis C virus
AFP: Alpha-fetoprotein
CT: Computerized tomography
HCC: Hepatocellular carcinoma.
